# What contributes to inappropriate antibiotic dispensing among qualified and unqualified healthcare providers in Bangladesh? A qualitative study

**DOI:** 10.1186/s12913-020-05512-y

**Published:** 2020-07-15

**Authors:** Papreen Nahar, Leanne Unicomb, Patricia Jane Lucas, Mohammad Rofi Uddin, Mohammad Aminul Islam, Fosiul Alam Nizame, Nirnita Khisa, S. M. Salim Akter, Emily K. Rousham

**Affiliations:** 1 Department of Global Health and Infection, Brighton and Sussex Medical School, University of Sussex, Brighton, UK; 2 International Centre for Diarrhoeal Disease Research, (icddr, b), Dhaka, Bangladesh; 3 School for Policy Studies, University of Bristol, Bristol, UK; 4 Paul G. Allen School for Global Animal Health, Washington State University, Pullman, Washington, USA; 5 Centre for Global Health and Human Development, School of Sport, Exercise and Health Sciences, Loughborough University|, Loughborough, UK

**Keywords:** Antibiotic, Antimicrobial resistance, LMIC, Informal healthcare, Prescription, Over-the-counter, Health provider, Health system, Pharmacy

## Abstract

**Background:**

Over-prescribing and inappropriate use of antibiotics contributes to the emergence of antimicrobial resistance (AMR). Few studies in low and middle-income settings have employed qualitative approaches to examine the drivers of antibiotic sale and dispensing across the full range of healthcare providers (HCPs). We aimed to explore understandings of the use and functions of antibiotics; awareness of AMR and perceived patient or customer demand and adherence among HCPs for human and animal medicine in Bangladesh.

**Methods:**

We used an ethnographic approach to conduct face-to-face, in-depth interviews with 46 community HCPs in one urban and one rural area (Gazipur and Mirzapur districts respectively). We purposefully selected participants from four categories of provider in human and veterinary medicine: qualified; semi-qualified; auxiliary and unqualified. Using a grounded theory approach, thematic analysis was conducted using a framework method.

**Results:**

Antibiotics were considered a medicine of power that gives quick results and works against almost all diseases, including viruses. The price of antibiotics was equated with power such that expensive antibiotics were considered the most powerful medicines. Antibiotics were also seen as preventative medicines. While some providers were well informed about antibiotic resistance and its causes, others were completely unaware. Many providers mistook antibiotic resistance as the side effects of antibiotics, both in human and animal medicine. Despite varied knowledge, providers showed concern about antibiotic resistance but responsibility for inappropriate antibiotic use was shifted to the patients and clients including owners of livestock and animals.

**Conclusions:**

Misconceptions and misinformation led to a wide range of inappropriate uses of antibiotics across the different categories of human and animal healthcare providers. Low awareness of antibiotic action and antibiotic resistance were apparent among healthcare providers, particularly those with little or no training and those in rural areas. Specific and targeted interventions to address AMR in Bangladesh should include educational messages on the rational use of antibiotics and how they work, targeting all types of healthcare providers. While tailored training for providers may increase understanding of antibiotic action and improve practices, more far-reaching structural changes are required to influence and increase responsibility for optimising antibiotic dispensing among all HCPs.

## Introduction

Global resistance to antibiotics among clinically important and commensal bacteria is increasing at an alarming rate, thereby threatening the effective treatment of infectious diseases [1, 2]. Antimicrobial resistance (AMR) has both health and economic implications, with increased costs of healthcare for humans and animals associated with resistant infections due to longer duration of illness, additional tests and the use of more expensive drugs. Overuse, incorrect and incomplete doses of antibiotics contribute to the development of resistance [2–4]. Antimicrobial resistance is a concerning health threat in low and middle-income countries (LMICs) where there is a shortage of qualified healthcare providers and heavy reliance on private providers, many of whom are unqualified [5].

In many South and Southeast Asian countries, antimicrobials are widely available as over-the-counter drugs purchased without prescription from registered or unregistered retail drug outlets. Excess use of antibiotics in livestock, poultry, and aquaculture is a further driver of antimicrobial resistance [6, 7]. A study in Vietnam found the proportion of antibiotics sold from pharmacies without a prescription was 91% in rural and 88% in urban areas [8] with low knowledge of antibiotics and antibiotic resistance among both customers and drug sellers [8, 9].

In Bangladesh, the scale of over-the-counter sales combined with the high number of retail outlets poses a considerable burden on regulatory authorities. Similarly, the limitations in human, technical and logistic capacity of the state render regulatory enforcement relatively weak. The presence of unregulated drug shops and a ‘pluralistic’ health system (government provision and health markets) compound this complexity [3]. In urban slum settlements in Dhaka, 75% of private sector healthcare is provided by pharmacies, informal providers or traditional doctors and only 20% is provided by qualified doctors [10]. In rural areas, the majority of healthcare providers are unqualified or semi-qualified [11].

Survey-based studies in Bangladesh among both qualified and unqualified healthcare providers (HCPs) show complex prescribing behaviours characterized by polypharmacy, high use of antimicrobials and almost non-existent generic prescribing [12–14]. Nationally, an estimated 63% of antibiotic prescriptions are from unqualified providers, and antibiotics are prescribed in nearly half (44%) of all consultations in primary health care [15]. Cross-sectional surveys in government health centres have reported 44% of outpatients receiving antibiotics [14] and, elsewhere, 71% of inpatients receiving an antibiotic [16]. Prescribers generally diagnose microbial infection based on signs and symptoms and select antimicrobials empirically rather than following standard treatment guidelines [17]. As the drug supply chain in the public sector fails frequently, people attending health centres are often directed to retail drug outlets, often unregistered, to purchase antibiotics [18]. The cost to patients is considerable, with studies showing that antimicrobials account for more than half of the total cost of prescriptions [13]. Moreover, the poor rely heavily on unqualified healthcare providers where misuse and overuse of antibiotics are common [19]. Qualified and unqualified providers alike are subject to aggressive marketing strategies of pharmaceutical companies [4, 17, 20, 21].

Despite meeting or exceeding millennium development goals in maternal and child mortality, universal immunisation and community-based health provision [22], the burden of infectious diseases in Bangladesh remains high and this is a barrier to continued health improvements. AMR has received increased attention in Bangladesh following the tripartite alliance of WHO, Food and Agricultural Organisation and the World Organisation for Animal Health [1] and subsequent development of the multi-sectoral national action plan (NAP) on AMR [23].

Progress against targets set in the National Action Plan on AMR is hampered by a lack of information on the socio-cultural dimensions of antibiotic prescription and use. There is a significant gap in knowledge around the dispensing behaviours of qualified and unqualified HCPs for antibiotics in low resource settings, both in humans and in livestock or animals. Humans and animals have closely shared environments in Bangladesh and the division between healthcare provision for humans and animals is less distinct, with some providers serving both sectors [24]. This study attempts to address this knowledge gap. The buying and selling process of antimicrobials is an important part of the ‘social life’ of antibiotics and is influenced by the awareness and understanding of providers about the function and use of antibiotics [25]. The knowledge, awareness and understanding of prescribers and providers about a particular medicine, therefore, is one of the components that plays a key role in the ‘social life’ of that medicine.

This study aimed to explore healthcare providers’ understandings of the use and functions of antibiotics; awareness of AMR, and perceived patient demand and adherence in urban and rural Bangladesh employing a One Health approach.

## Methods

As part of a larger qualitative study on the pathways of use of antibiotics within household and among healthcare providers [26] we report the ethnographic component conducted with community-based HCPs in human and animal medicine. The study took place in a rural area of Mirzapur district (*upazila*) and in the urban town centre of Tongi in Gazipur district from July 2017 to January 2018. The study adopted a One Health approach by examining providers for both human and animal care including registered doctors and veterinarians; qualified healthcare practitioners, and providers with no formal qualifications. HCPs were identified who dispense or prescribe allopathic medicine (biomedicines) across the human and animal sectors. Considering the context of medical pluralism within the biomedical sector in South Asia [27], we included biomedicine providers from the formal and informal sectors. Since the focus was on allopathic providers, we excluded the traditional sector or alternative medicine practitioners, although they may also prescribe or dispense medicines at times.

Based on the literature and the range of qualifications available in Bangladesh we identified four categories of healthcare provider for human and animal medicine (see [26]. Namely: ***Qualified practitioners*** were those with formal degrees in veterinary medicine, human medicine or pharmacy. ***Auxiliary medical practitioners*** were defined as persons with 1–4 years of medical-related training i.e. a diploma in medicine or health professions such as nursing, medical technology, or family welfare visitors. These professionals prescribe or sell from a restricted list (provided by the government) that includes some antibiotics. No equivalent auxiliary professionals were found within the veterinary medicine sector under this category. ***Semi-qualified providers*** were defined as having less than 1 year of health-related training and selling medicines or providing prescriptions (verbal or written) for humans or animals. ***Unqualified providers*** were defined as those without any health-related training, who sell medicines, assist the shop keeper or provide prescriptions (verbal or written) for humans or animals.

The interview guide was developed based on reviewing the relevant literature and through consultation with the full research team and field researchers and is available in English and Bengali [28]. In interviews, we explored the level of understanding of antibiotics; the meaning, use and action of antibiotics as well as how antibiotic resistance occurs. We piloted the guide with seven participants from across all categories of provider. Pilot interviews were not included in the data for analysis but were used to refine the interview guide along with feedback from the investigating team. An extensive 14-day classroom and field training workshop on qualitative research methods was carried out by the first author, a medical anthropologist with more than 15 years’ experience. The field team comprised one female and two male researchers employed by icddr,b (NK, MRU, SMSA), all with relevant Masters’ degrees and extensive fieldwork experience (ranging from 4 to 12 years). Oversight of fieldwork, recruitment, sample composition was carried out by the research manager and research team.

Ethical approval for the study was granted by the Institutional Review Board at icddr,b (PR-16100) and Loughborough University (R17-P081). Prior to starting interviews, three field staff and the lead investigator visited key personnel and government authorities in the two communities to explain the purpose of the study and seek local permissions. Before data collection, the first author and field team visited the two districts for site selection and rapport building and familiarisation with the community people and leaders. Pilot data collection was also carried out in these two selected sites. Through these processes the researchers built relationships with the study community. Recruitment took place by the researchers and community leaders approaching respondents directly. Healthcare providers were provided with written study information in Bengali which was read aloud to them and they were given an opportunity to ask questions before signing a written informed consent form in Bengali. We recruited respondents using purposive sampling via snowball sampling [10, 21] and aimed to include the range and variety of qualified, semi-qualified and unqualified providers at the community healthcare level. Recruitment continued until the data reached the point of saturation, which was judged against the sample targets and in discussion with the field researchers to establish when they felt they were no longer achieving new material.

Face-to-face in-depth interviews took place in the healthcare providers’ place of work (i.e. clinic, drug shop, consultation chamber or government health facility). The underpinning theoretical and methodological approaches were grounded theory and ethnography [29]. A thematic analysis was conducted using a framework analysis process [30]. The duration of interviews was typically 1 h. There were no refusals but one participant (unqualified HCP) did not complete the interview and was therefore excluded from the analysis. Field notes were written alongside the audio-recording and referred to during transcription and data analysis.

Interviews were audio-recorded and transcribed anonymously in Bengali. Following interview transcription, a third of interviews were translated to English and these were used to develop a draft framework. Framework analysis involves coding data into a framework, a process known as charting [31]. This coding list comprised both inductive and deductive codes; some themes were identified in advance using the interview guide and the remaining codes were derived from the data [29]. The draft framework was shared with the wider study team and all worked together to iterate versions until a final version was agreed. After identifying and agreeing codes, the Bengali-speaking researchers charted the interviews and conducted the first stage analysis in Bengali (PN, MRU, FN, NK, SMSA). Fifty percent of the interviews were double coded during the first stages of analysis with the team working together. For those interviews not translated in full, the bilingual research team members coded Bengali transcripts into framework charts, and these charts were then translated to English for further analysis across the team. The first author was involved in every stage of data analysis both in Bengali and English. The quality of interview and analyses were checked by the first author on a regular basis.

Table 1 presents the number of providers in each category and the total number of interviews. We recruited 12 qualified providers; 7 auxiliary medical practitioners; 14 semi-qualified providers, and 13 unqualified providers. Of the total sample, 25 participants provided medicines for humans; 12 provided medicines for animals, and 9 provided medicines for both humans and animals. In the latter category, all participants were semi-qualified or unqualified (see Table 1).

**Table 1:** Composition of the sample of in-depth interviews according to the four categories of healthcare provider

Category	Type of provider	Rural	Urban
Qualified Graduate Practitioner	Public and private; human and veterinary medicine practitioners	6 (4 human, 2 veterinary)	6 (4 human, 2 veterinary)
Auxiliary medical practitioner^a^ (1–4 years training)	Healthcare workers, paramedics, family welfare visitors, health assistant	4	3
Semi-qualified healthcare provider (1–12 month of training)	Retail drug shop owner or seller	6 (2 human, 2 animal, 2 both human and animal)	8 (4 human, 2 animal, 2 both human and animal)
Unqualified healthcare provider (no training)	Retail drug shop owner or seller	7 (2 human, 2 animal, 3 both human and animal)	6 (2 human, 2 animal, 2 both human and animal)
**Total**		**23**	**23**

^a^Provider in human medicine only, no veterinary equivalent

## Results

### ‘Antibiotic, a medicine of power’

Among all the categories of providers, antibiotics were associated with the symbol of power. It was evident from their responses that they differentiate antibiotics from other medicines by emphasizing their ‘power’ to combat diseases. When they talk about antibiotics, they always refer to them as strong and powerful medicines.

*“.....an antibiotic is powerful medicine and it works against bacteria. I give antibiotics when patients would not get well by the normal (other) medicines.”* (Rural unqualified provider for humans)

Although the qualified providers did not use the term ‘power’ while talking about antibiotics, they thought this was the general perception among their patients. Qualified doctors mentioned that the general population sees antibiotics as medicine of power. As one doctor said,

"*They (patients) don't know what an antibiotic is, but for children, they say to us, ‘please give me that medicine that is a powder and needs to be mixed with water; that is very powerful’. You know they only understand that this is powerful but don't know what an antibiotic is. But they know if they don't get that medicine then patients would not get well, and they think it is a very effective medicine for getting well”* (Urban qualified provider for humans)

Most of the unqualified providers think antibiotics start working by reducing fever, so they use antibiotics in cases when the fever has not subsided after taking other medicines,

*"If the fever is not cured within three to five days then we prescribe Zimax (azithromycin) or Ciprocin (ciprofloxacin) antibiotics for five to seven days for patients. ...It seems that most of the patients get cured by taking antibiotics"* (Rural unqualified for both human and animal).

A few auxiliary providers mentioned that antibiotics give quick results, therefore, they gave these mainly to children in response to demand from mothers. According to the providers, the demand for antibiotics from the customers come in various vocabularies. Some demand a powerful medicine, some ask for a medicine that would cure the disease in 2 days, all these expressions have an implicit meaning of seeking antibiotics. On the other hand, some ask explicitly for antibiotics by name e.g. *Ciprocin*.

### ‘Antibiotic, a medicine that works against all diseases’

Despite an awareness of the powerful actions of antibiotics, most of the providers had a poor understanding of their function. When explored, a gross lack of awareness about the action of antibiotics was apparent, particularly among the unqualified, semi-qualified and auxiliary providers. Most thought an antibiotic was the strongest medicine among all by way of its functions and quick actions. There was a common belief among all except fully qualified providers that antibiotics work against all kinds of diseases, ranging from viral and bacterial infections. Antibiotics were not thought to work for non-communicable conditions. Several unqualified and semi-qualified providers thought that antibiotics can kill viruses. Due to the discrepancy in understanding the function of antibiotics, inappropriate use of antibiotics was widespread. For example, a provider expressed how useful antibiotics are as a medicine for any disease,

“*Antibiotics are effective because they kill the germs. They are effective for fever, typhoid, viral fever, tuberculosis etc. They are also effective for measles. You can take them for diarrheal diseases, cough and for minor injury”* (Urban unqualified for human).

Another provider had a similar view, *“The function of an antibiotic is that it works against the viral or bacterial infections of our body. It can be used as a vaccine (it kills the germs and protects the body for some time). So, I think that its function is fully different. Its work is different from other medicines.”* (Urban semi-qualified for human).

Like the informal providers, a few auxiliary providers also believed that antibiotics are effective against many diseases including those caused by viruses.

"*Usually antibiotics destroy the virus and bacteria, … ...The task of antibiotics is to destroy the virus”* (Rural auxiliary for human).

However, no such idea was reported among the qualified providers.

### ‘Price and power of antibiotics’

The price of antibiotics matters in terms of judging its power for both the providers and the patients. Qualified providers had knowledge and understanding of the generations of antibiotics. However, they also acknowledged that patients judge an antibiotic by its price. They thought that from the patients’ perspective, antibiotics are more expensive compared to generic medicines and amongst antibiotics, the pricier ones are the most powerful. As elaborated,

"*They don't know if it is antibiotic or not, they only know it is expensive medicine. … They actually feel they don’t need to know the name of medicines or whether it is an antibiotic or not, or why this specific medicine is given, they just want to know the price.*" (Urban qualified for human).

Most of the unqualified providers did not have a clear idea about the different generations of antibiotics. The idea of generation was expressed as a ‘group’ of antibiotics. They also did not know which antibiotics belonged to each generation of antibiotics, rather they had an indigenous way of differentiating generations. To them, the more expensive the antibiotic, the more powerful it is. They consider expensive antibiotics as always being of an advanced generation. A rural unqualified provider who dispenses medicine for both humans and animals said,

“*The strength of an antibiotic depends on its price. Powerful antibiotics can cure fevers instantly. They can also help cure infections, colds, coughs and toothaches*”.

To them, an expensive antibiotic has the capacity to overpower any disease, particularly the ones that are unexplained.

### ‘Half antibiotics’

The unqualified and auxiliary providers had developed certain vocabularies surrounding antibiotics while talking about their action. For example, they differentiated between ‘full’ and ‘half’ antibiotics. Full antibiotics referred to the most powerful ones, while those they considered less effective were referred to as half-antibiotics. For the providers, the association between strength and power of an antibiotic was so integral that even stronger or more powerful non-antibiotic medicines are understood as antibiotics. For example, for some unqualified providers, paracetamol (also known as acetaminophen) containing drugs such as ‘Napa extra’ (paracetamol plus caffeine) is considered a ‘half’ antibiotic. Some also used the terms ‘full’ and ‘half’ to describe first and subsequent generations of antibiotics.

“*Usually antibiotics destroy the virus or bacteria … Half-antibiotics are Napa Extra, Amoxicillin … and when we need stronger antibiotics we give Cefuroxime and so on”* (Rural unqualified for human).

*“ … A half antibiotic is Napa Extra (paracetamol/acetaminophen plus caffeine), Fimoxyl (amoxicillin), these are normal medicines. When we give higher antibiotic then we give Roxim (Cefuroxime), Cef-3 (cefixime). ...If they (the patients) miss taking their antibiotic medicine during a course then they have to take that course again and complete the course. They have to take for the second time, a higher antibiotic. ...full antibiotic medicine means a higher antibiotic which destroys the virus*" (Rural auxiliary for human).

### ‘Antibiotics as preventive medicines’

Like other misconceptions about antibiotics, a number of unqualified and semi-qualified providers thought that antibiotics can prevent disease in addition to curing it. Significantly this belief of antibiotics being a preventative medicine also prevailed among auxiliary providers. Some even thought that antibiotics work as a vaccine. These ideas were more common among rural semi-qualified and auxiliary providers than their urban counterparts. As one provider said,

*“Antibiotics create an antibody so that other germs do not attack us. So, antibiotics increase the power of disease prevention.* (Rural auxiliary for human).

In general, the urban unqualified and semi-qualified providers had relatively better knowledge than their rural counterparts about the functions and generations of antibiotics, as well as differential use of antibiotics for specific diseases. Similarly, urban semi-qualified providers had better knowledge than unqualified providers. One urban semi-qualified provider for humans said,

“*I give antibiotic to those who suffered from fever and already consumed/took paracetamol (acetaminophen) but the fever has not been cured. I start with Amoxicillin or Ciprocin (ciprofloxacin) first and observe for five days then go for more powerful antibiotics”.*

Semi-qualified providers appeared to take a trial and error approach in their provision of antibiotics. One semi-qualified provider said,

“*For example, I give the first generation to ten patients and if I see that eight out of them are not getting cured then I start the third generation. I will then not use the first generation anymore...”* (Rural semi-qualified for both human and animal).

### ‘Preference for advanced generation antibiotics’

Although the informal providers did not have a full understanding of the generations of antibiotics, they figured this out through other means. Many a time they did this through the difference in price; learning by prescribing as mentioned earlier or by following a doctor’s prescription. Whatever the means of identifying antibiotic generations, it was evident that providers had a tendency to prescribe antibiotics of advanced generation. An urban semi-qualified provider for humans said,

"*Nowadays we are using high-power antibiotics for all diseases, arthritis-fever or loose motions, headaches, and for various gynaecological problems*”.

Also, an urban unqualified provider for human medicine said:

“*Nowadays we skip directly to fourth generation antibiotics, the first-generation amoxicillin is not effective anymore.*”

The unqualified providers for animals also dispensed antibiotics for all kinds of animal diseases. An unqualified animal health provider stated,

*“I usually provide doxycycline, oxytetracycline, tylosin or a combination of multiple antibiotics. These types of antibiotics help to cure a cold, cough and fever of livestock”.*

However, unlike unqualified providers, the auxiliary providers had some knowledge about different generations of antibiotics which they obtained through their medical training given by the government health sector.

*“Amoxicillin and doxycycline are primary antibiotics; the second generation is a high-level antibiotic. Azithromycin is a second-generation antibiotic which is a higher antibiotic, if any patients get a severe cold then we use it but at the union (local) level the government does not provide it. We were told in our training (certificate training) that we can give only primary treatment based on signs and symptoms if there is anything that we do not understand then we refer them to the hospital... By consuming high [generation?] antibiotics children get well quickly. Mothers want their child's quick recovery, that’s why we give them.”* (Rural auxiliary for humans).

In contrast to the other groups, the qualified providers generally had a good understanding of the use, appropriate dose and actions of antibiotics. As one qualified provider for animals said:

“*If, for example, I diagnose that the animal disease is Black Quarter, I know that this disease is occurring by the infection of a Gram-positive bacteria; in that case, I can easily choose to prescribe penicillin. Again, if I diagnose a Gram-negative bacteria infected disease then I try to give treatment with the gentamicin group of antibiotics or specific drugs which will respond to the Gram-positive and negative bacteria.*"

On the other hand, even though qualified providers for animals had knowledge about the generation of antibiotics, they did not always prescribe these rationally. As one qualified urban animal health provider said without much consideration that he immediately prescribes third generation antibiotics:

*“Antibiotics work against Gram-positive organisms, gangrene and mastitis diseases. Most of the time I prescribe third generation antibiotics straight away and I mix a combination of antibiotics for cattle”.*

Misconceptions about the use, appropriate dose and function of antibiotics particularly among unqualified, semi-qualified and auxiliary providers seem to be similarly prevalent in rural and urban settings.

### ‘What is Resistance?’

An important component of this study was to explore the understanding among providers about antimicrobial resistance. Providers of antibiotics for humans in rural areas showed a varying degree of knowledge about resistance, with variation within and between different categories of providers. Among unqualified, semi-qualified and auxiliary providers some were well informed about the term and causes of antibiotic resistance whilst others were completely unaware.

One rural unqualified provider for humans said,

“*I have not heard about resistance. But, I think that if someone does not take antibiotics correctly, then the disease condition will get worse day by day*.”

One auxiliary provider also had vague ideas about resistance and said,

*“Sometimes the antibiotic’s power gets diluted in the body then it does not work against the disease. Then the patient keeps on suffering from the disease. Then we need to give more powerful antibiotics”.*

One striking finding was that many providers, when asked about antibiotic resistance, mistook this as the side effect of antibiotics. One semi-qualified provider said, antibiotics can be harmful to teeth in growing children. Another one said antibiotics can damage children’s cartilage.

One urban auxiliary provider for humans mentioned antibiotics can cause allergy and scabies as a side effect. When asked about resistance one urban unqualified provider said,

*“After having Azithromycin, a few patients complained that they had swelling in the body. Although I did not find any reactions in any other antibiotics like this.”*

Similarly, a semi-qualified provider for animals talked about the side effects of antibiotics for animals when asked about resistance:

*“Sometimes after having antibiotics, swelling or infection appears on the whole body and the cow can’t stand up and feels a headache. That is resistance”.* (Rural semi-qualified for animal).

A respondent said, “*Antibiotics are harmful medicines. If anyone does not take the full course of medicine, then it will not be working exactly. For that reason, diseases may increase more in future. Suppose in the future a patient may not be able to walk, talk or take food properly* (meaning disabled) *due to taking irregular antibiotics”.* (Rural unqualified for both human and animal).

However, the providers who were informed and aware of AMR related it to irregular consumption, incomplete doses, or the overuse of antibiotics. An auxiliary urban provider for humans also pointed out the dangers of inappropriate use of antibiotics,

“*If any patient doesn’t complete the full courses of antibiotics, then in future the medicines will not work accordingly. I always tell the patients ‘if you do not face any problem then still please don’t stop it after consuming one or two days later’. Because if someone doesn’t complete the antibiotic course properly over several occasions then that drug becomes resistant.*”

One rural semi-qualified provider for humans also mentioned how failing to comply with the appropriate dose of antibiotic can cause serious consequences,

*“Antibiotics are a dangerous medicine. If someone does not complete the full course, it will not work properly. In which case, the disease may become even more severe.”*

A qualified provider for humans used a metaphor to explain resistance. He said,

“*Our human body is like a battlefield, antibiotics are the main missiles. When we invent any new drugs, it is like we invented a new missile against the germs. If the opposition party gets any information before then they will create a protection system like anti-missiles. So, when antibiotics are not used fully then bacteria will create an antibody against antibiotics and it will not work against that disease or germs. These are all antibiotic resistance mechanisms*”. The qualified provider points out how overuse of antibiotics can cause resistance.

### ‘Responsibility for resistance’

Despite the varied knowledge of AMR, all providers showed concern about antibiotic resistance. When asked about how to prevent this risk the providers usually shifted the responsibility to one other. For example, a qualified provider thought that to prevent antibiotic resistance all practices by the unqualified providers should be stopped because they are incompetent:

*“Number one would be that without a prescription no pharmacy outlet should be allowed to sell their drugs. Without a doctor’s consultation, no one should take medicines, we should follow these two steps then resistance will stop. Rural practitioners [‘Unqualified providers’] should stop their practice because they have training for only two months and become doctors who give the wrong treatment to the patients. You will see that they prescribe third or fourth generation antibiotics like cefuroxime, for any diseases, they use all broad-spectrum antibiotics that we keep as a reserve for later use.”* (Rural qualified provider for human).

On the other hand, a rural unqualified provider for animals saw the problem within the qualified providers, who he thinks should be controlled.

“.....*a farmer depends on the doctor and the doctor has a liaison with pharmaceutical companies who push them to sell antibiotics, so a policy should be there to control the qualified doctors and pharmaceutical companies in their control of the overuse of antibiotics or drugs.”* (Rural unqualified for animals).

All categories of providers, irrespective of their location, blamed patients for contributing to antibiotic resistance by not following instructions and failing to adhere to the course of drugs as directed.

“*Patients do not complete the course. They take an incomplete dose and become resistant. They take it for one day or two days only*”. (Urban semi-qualified for both humans and animals). Also, “*If you count it then you will find only 20% to 30% of people follow the instructions, the majority do not follow the instruction and do not consume it [antibiotics] properly.”* (Rural auxiliary for humans).

Reasons for non-compliance by the patients were explained as the inability to afford the full course of medicine; illiteracy and ignorance of the patients or stopping the medication due to feeling better. One qualified provider for humans said,

*“Suppose we have prescribed medicines for ten days but many patients consume it just for five days because they do not have enough money to buy the full course.”*

Considering the patients’ ability to afford medicines the unqualified providers sometimes deliberately gave a partial dose of antibiotics instead of the full course.

“*… sometimes poor men such as rickshaw pullers visit me with a severe fever for over 7 days, since they cannot afford to visit the private and qualified (MBBS) practitioners. Then, as per their request, I prescribe them a few antibiotic capsules.”* (Urban unqualified provider for humans).

The providers thought patients do not adhere to antibiotic courses in some cases because they do not understand the instructions from the providers or read the instructions written on the medicine packet due to their illiteracy.

“*Many of them (patients) don’t understand that they are taking antibiotic due to lack of education. Even here in this slum many of them are illiterate, I mean, they are all slum dwellers. They are not conscious and maybe they don’t know what an antibiotic is*”. (Urban qualified provider for humans).

Some providers stated that patients do not continue medicine once they or their animal start feeling better or if they experience any side effects.

*“Sometimes it is seen that after getting some antibiotics, the cattle become healthy. Then they do not provide any medicine to their cattle. But it has been observed that the disease appears again. ...* “. (Rural semi-qualified for animals). Also,

“*While they consume (antibiotics) if they feel vomiting or swelling then they do not continue the drugs. Sometimes they stop antibiotics suddenly due to some side effects, but they do not understand why it is happening. A person may feel unwell by consuming another drug that they may be taking but their first action is to stop the antibiotics.*” (Rural auxiliary for humans).

The providers emphasized the need to educate patients through various means. For example, a qualified provider suggested using mass media to raise awareness:

*In our country we have two popular media, television and radio, for building mass awareness - we can use these two media. In every hospital, we need a counsellor who will give advice to the patients about drug adherence.” (Rural qualified provider for humans).*

It is interesting to note that the providers do not take any responsibility for creating the problem of antibiotic resistance.

## Discussion

This study provides novel insights into the supply side of antibiotics at the community level in rural and urban Bangladesh. Antibiotics were considered powerful medicines that give quick results and work against almost all diseases including viruses. Awareness and understanding about antibiotics and resistance among providers reflected the varying level of qualifications as well as the location of practice (urban vs rural). Urban providers tended to have a better understanding of the correct course duration and generations of antibiotics than their rural counterparts. Urban providers were also better informed about the function of antibiotics. While several factors could contribute to this difference, we found that providers in urban areas had greater exposure to, and interaction with, medical representatives from pharmaceutical companies compared to providers in rural areas which could be one contributing factor. Pharmaceutical company representatives have been highlighted as important sources of knowledge on antibiotics among informal providers in India [32] and are the only routine source of information for unqualified providers in Bangladesh [33].

The price of antibiotics was equated with power such that expensive antibiotics were considered the most powerful medicines. Antibiotics were also seen as preventative medicines. The semi-qualified, unqualified and auxiliary providers in the study showed some gross misconceptions about the use, function and appropriate dose and duration of antibiotics. Although auxiliary providers would be expected to have fewer misconceptions than semi-qualified or unqualified providers, they too demonstrated vague knowledge about antibiotics and AMR. This important group of healthcare providers may have been overlooked in efforts to improve antibiotic stewardship [33].

While some providers were well informed about antibiotic resistance and its causes, others were completely unaware. Among providers who claimed that they were aware, many mistook antibiotic resistance as the side effects of antibiotics, both in human and animal medicine. Qualified providers had good awareness and understanding of antibiotic resistance, yet some were found to prescribe antibiotics irrationally, for example, by providing third-generation antibiotics as first-line therapy. A quantitative study in India also demonstrated a dissonance between knowledge and practice with qualified providers [32]. Mixed providers for human and animal medicine were more common in the rural site which could contribute to the use of human medicines for animals.

All providers who showed awareness of antibiotic resistance thought this was a concern, but they considered that this was due to irresponsible antibiotic use among patients and customers, including the owners of livestock and animals. Most strikingly, none of the HCPs from the formal or informal sectors acknowledged that their behaviour may contribute to AMR, in keeping with other studies [34]. All blame was shifted to the patients and clients. Patients and clients were also blamed for being ignorant about AMR, which the providers believed was the root cause of the problem.

A potential limitation of this study is that we focused on biomedicine providers and did not include traditional medicine providers or healers who may have added further perspectives on antibiotic understandings and use. Strengths of the study include the perspectives gained from a wide range of providers, especially unqualified providers who play a key role in antibiotic access. Potential bias within the study was addressed using qualitative methods of triangulation across the different data sources and methods of collection [29]. Qualitative studies aim to provide a contextualized understanding of human experience and produce a result that is transferable rather than attempting to be generalizable as with quantitative studies [35].

The emergence of antibiotic resistance can be examined according to the factors determining antibiotic use, overuse, and misuse [2, 36]. Our study focuses on the supply side of antibiotic use, which is critical in a pluralistic healthcare system. Tomson and Vlad [2] argue that antibiotic resistance should be addressed from a health systems perspective and that *human resources* for health is the key to understanding the supply-side factors driving AMR. The pluralistic health service provision and weak regulation of prescription-only medicines, coupled with poor awareness of AMR among providers, greatly hampers the supply-side competency of Bangladesh health services. Of note, however, the providers themselves did not relate the problem of antimicrobial resistance to competency within the supply side. Rather, interviewees placed blame on other types of HCPs or on the demand side, which they characterised as misconceptions and non-compliance among the patients. This study reveals how multiple stakeholders from the supply and demand side influence the provision of antibiotics. This echoes previous work highlighting that antibiotic prescribing is a complex process influenced by factors affecting all the actors involved: physicians, other healthcare providers, the healthcare system, patients and the general public [37].

Interestingly, household interviews revealed a heavy reliance of patients and consumers on unregulated retail drug outlets for both antibiotics and healthcare advice [18]. Retail drug outlets continue to play an essential role due to failures in the drug supply chain for public sector providers; long waiting times in government facilities and the high cost of private qualified providers [18]. The critical role of informal or unqualified healthcare providers for poorer households is a widely recognised pattern for multiple health issues [27, 38, 39].

The Bangladesh NAP [23] includes plans for awareness raising, advocacy, communication and developing continuous professional education curriculum across the ‘One Health’ spectrum. To address this first strategic area, it is important to know the country-specific situation of community healthcare provision and the knowledge of healthcare providers at the grassroots level. In such settings, efforts to reduce AMR will more likely come from empowering and educating unqualified providers and consumers [3]. Awareness raising among semi-qualified and unqualified providers is also important. Unorganised markets for antibiotics have, on the one hand, enabled people to treat infections and reduce mortality but, on the other hand, contributed to the overuse of antibiotics and behaviours likely to encourage the emergence of resistance [3].

## Conclusions

The study reveals that misconceptions and misinformation lead to a wide range of inappropriate uses of antibiotics across different categories of human and animal healthcare providers. We highlight important areas for reducing misconceptions around antibiotics among the key actors in healthcare provision. Our findings suggest that specific and targeted interventions to address AMR in Bangladesh should include educational messages on the rational use of antibiotics and how they work. Providers and consumers should be informed that antibiotics do not work for all diseases. Awareness raising around antimicrobial resistance needs to create a better understanding of both antibiotics, and resistance to antibiotics. Such interventions need to target qualified and unqualified providers across human and animal healthcare.

More far-reaching structural changes within the health system are required to influence and increase responsibility for optimising antibiotic dispensing among all HCPs. This paper has clear policy implications that demonstrate the need for the National Action Plan on AMR to increase advocacy around appropriate antibiotic prescribing particularly among less qualified and unqualified providers.

## Data Availability

Anonymised original data from the study can be accessed via Loughboorugh University data repository at: http://doi.org.10.17028.lboro.8953118.v1 .

## Abbreviations

AMR: Antimicrobial resistance
HCP(s): Healthcare provider(s)
NAP: National action plan on antimicrobial resistance
